# Therapeutic effects of vitamin D and omega-3 supplementation on HFHS diet-induced endothelial dysfunction in Wistar rats

**DOI:** 10.1016/j.crphar.2025.100240

**Published:** 2025-11-29

**Authors:** Dylan Le Jan, Sandrine Destrumelle, Chantal Thorin, Jean-Claude Desfontis, Eric Betti, Mohamed Yassine Mallem

**Affiliations:** a Nutrition, PathoPhysiology and Pharmacology (NP3) Unit, Oniris, 101 Rte de Gachet, 44300, Nantes, France; b INRAE Oniris, UMR 703, PanTher, APEX, 44307, Nantes, France

**Keywords:** Obesity, Endothelial dysfunction, Vitamin D, Omega-3 fatty acids, High-fat high-sugar diet, Vascular health

## Abstract

**Background:**

Obesity impairs cardiovascular health through endothelial dysfunction, which is exacerbated by high-fat high-sugar (HFHS) diets through mechanisms involving chronic inflammation, oxidative stress, and insulin resistance. Nutritional interventions, specifically vitamin D (VD) and omega-3 fatty acids (ω3), have emerged as potential therapies to improve endothelial function and mitigate cardiovascular risks associated with obesity.

**Objective:**

This study investigated the effects of VD, ω3 and their combination on endothelial dysfunction induced by an HFHS diet in Wistar rats.

**Methods:**

Sixty-four male Wistar rats were fed either a Standard (S) or HFHS diet for 26 weeks. After 13 weeks, rats were supplemented with VD (600 IU/kg/day), ω3 (300 mg/kg/day), both (VD/ω3), or a control (C) for an additional 13 weeks. Endothelial function was assessed using aortic ring assays, focusing on acetylcholine (ACh)-mediated endothelium-dependent vasorelaxation, phenylephrine (Phe)-mediated vasoconstriction, and insulin responsiveness.

**Results:**

VD and/or ω3 supplementation effectively improved ACh-mediated vasorelaxation and counteracted HFHS-induced endothelial dysfunction. VD enhanced insulin-mediated vasorelaxation, while ω3 showed a non-significant trend towards improved Phe-mediated vasoconstriction.

**Conclusion:**

VD and ω3 supplementation, alone or in combination, significantly improved endothelial function and mitigated the adverse effects of an HFHS diet. The combination did not show clear additive effects. These findings suggest their potential as therapeutic strategies for managing obesity-related cardiovascular issues.

## Highlights

• A high-fat high-sugar diet induces endothelial dysfunction in Wistar rat aortas.
• Vitamin D and omega-3 fatty acids improve endothelium-dependent vasorelaxation.
• Omega-3 shows a trend toward improving hypocontractility.
• Vitamin D enhances insulin-mediated vasorelaxation, improving insulin sensitivity.
• Further research is needed to assess vitamin D and omega-3 fatty acids synergy.

## Graphical abstract

**Figure:**
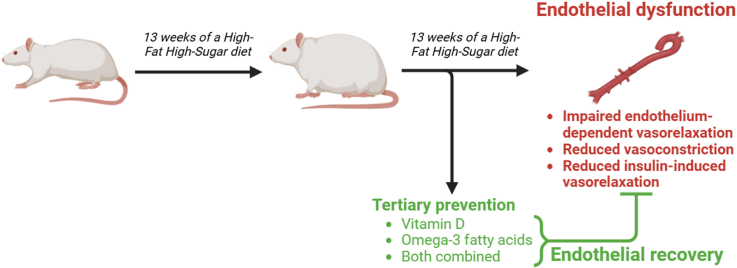

## 1 Introduction

In obesity, endothelial dysfunction arises from chronic inflammation, oxidative stress, and metabolic dysregulation. Excessive fat accumulation promotes these mechanisms, impairing endothelial nitric oxide synthase (eNOS) activity and reducing nitric oxide (NO^•^) bioavailability (Avogaro et al., 2008; Chen et al., 2018; Wang et al., 2022). Moreover, oxidative stress contributes to peroxynitrite (ONOO^−^) formation, depleting NO^•^ and oxidizing tetrahydrobiopterin (BH_4_), an essential eNOS cofactor (Cai and Harrison, 2000). These processes worsen insulin resistance and endothelial dysfunction (Hotamisligil, 2006; Nieto-Vazquez et al., 2008), leading to impaired vasodilation, increased inflammation, and elevated cardiovascular risk (Engin, 2017; Konukoglu and Uzun, 2017; Sitia et al., 2010).

Nutritional strategies have shown promise in mitigating obesity-related cardiovascular alterations.

Among them, omega-3 fatty acids (ω3) and vitamin D (VD) have demonstrated potential in protecting endothelial function. ω3 fatty acids, including eicosapentaenoic acid (EPA) and docosahexaenoic acid (DHA), possess anti-inflammatory and antioxidant properties (Wall et al., 2010). In humans, ω3 improves endothelial function, as evidenced by increased flow-mediated dilation in brachial artery (Wang et al., 2012). Preclinical studies show improvements in endothelial function in both *in vitro* and *in vivo* high-fat diet (HFD)-induced obesity models. For example, ω3 supplementation restored impaired flow-mediated vasodilation in mesenteric arteries by activating TRPV4 channels (Zhu et al., 2021). Additionally, a flaxseed oil-enriched diet alleviated vascular inflammation and endoplasmic reticulum stress in obese mice (Moura-Assis et al., 2018). Moreover, ω3 supplementation reversed endothelial dysfunction, enhancing NO^•^-mediated relaxation and decreasing oxidative stress (Farooq et al., 2020), positioning ω3 as a promising intervention for endothelial health.

VD deficiency has been linked to increased cardiovascular risk, partly due to its impact on inflammation and endothelial function (Mozos and Marginean, 2015). VD supplementation reduces inflammation and oxidative stress, key contributors to obesity-related vascular dysfunction (Renke et al., 2023). In diabetic models, VD mitigates endothelial dysfunction and supports cardiovascular protection (Said, 2022). However, its impact in obesity remains controversial, with some studies reporting no significant cardiovascular benefits (Veloudi et al., 2016), or VD inefficacy against obesity-related metabolic complications (Wamberg et al., 2015). Despite this, VD's potential to counteract endothelial dysfunction and oxidative stress warrants further exploration.

Combining VD and ω3 may represent a promising strategy to counteract obesity-related endothelial dysfunction. While ω3 are recognized to improve endothelial function by promoting NO^•^ production and reducing inflammation, VD has also been linked to reduced inflammation and improved vascular function. Based on their complementary biological actions, co-supplementation with VD and ω3 was selected. Both nutrients influence inflammation and insulin sensitivity albeit through distinct molecular pathways. VD primarily through the VDR and ω3 through PPARs and GPR120. Combining them allows targeting multiple mechanisms simultaneously, potentially enhancing efficacy against obesity-related vascular dysfunction. Previous studies in rodents and humans suggest that such combinations may produce additive or synergistic effects across different conditions, including cancer, autism, and diabetes, although results remain variable and further investigation is warranted (Basyreva et al., 2021; Dyck et al., 2011; Infante et al., 2020; Refaat et al., 2022).

However, their combined effects in the context of obesity remain unexplored. To the best of our knowledge, the combined effects of VD and ω3 have never been explored in this context. Investigating their synergistic potential is essential for gaining a deeper understanding of how obesity affects vascular function, which could ultimately lead to more effective strategies for managing obesity-induced endothelial dysfunction.

This study investigated the therapeutic effects of VD and/or ω3 supplementation on obesity-related endothelial dysfunction in Wistar rats, focusing on the thoracic aorta. We hypothesized that these supplements, individually or combined, would enhance endothelial function by attenuating inflammation and oxidative stress, thus alleviating vascular impairments associated with HFHS diet-induced obesity.

## 2 Methods

### 2.1 Reagents and chemicals

Acetylcholine chloride (Ach; A6626), phenylephrine hydrochloride (Phe; P6126), insulin (Ins; I2643), tempol (581500), L-arginine (L-Arg; A5131), BH4 (T4425), aminoguanidine (AG; 396494), were purchased from Sigma-Aldrich (St. Louis, MO, USA), while potassium chloride (KCl; 131494.1211) was purchased from Grosseron (Couëron, France) and Wortmannin (Wort; 1232) was purchased from Tocris (Bristol, United Kingdom). All chemicals were of analytical grade and prepared in accordance with the manufacturer's instructions.

### 2.2 Animal housing and nutritional intervention

A total of sixty-four male Wistar rats, aged 8 weeks, were procured from Janvier Labs® (Le Genest-Saint-Isle, France) and subjected to a one-week acclimatization period. The housing conditions met European standard ETS 123 requirements, including a 12-h light/dark cycle, a controlled temperature of 22 ± 2 °C, and a humidity level of 50 %. All experimental procedures were conducted in accordance with established best practices and the 3R principles (Replacement, Reduction, Refinement). Ethical approval was obtained from the Pays de la Loire ethics committee with the French research ministry authorization (APAFIS#33784-2021112413036973v3).

Upon arrival, the rats were randomly assigned to the two dietary groups (Standard and HFHS, n = 32 per group). After 13 weeks, each dietary group was further randomly subdivided into four treatment subgroups (Control, VD, ω3, VD/ω3; n = 8 per subgroup). This random allocation ensured unbiased distribution of animals across groups for the subsequent supplementation interventions. Each subgroup contained 8 rats, a sample size selected to balance the ability to detect subtle physiological and vascular differences with the principle of minimizing animal use. This choice was further supported by our team's previous experience in similar experimental settings.

Initial body weights were comparable across all groups, with no significant differences observed, confirming an unbiased baseline distribution of animals.

The rats were fed different diets for 26 weeks. One group received a standard diet (S; 3.84 kcal/g) from 3430PMS10 Serlab® (Montataire, France), while the other group was given a HFHS diet comprising pellets (4.73 kcal/g) from D12451 Research Diets® (Lynge, Denmark) and sweetened condensed milk (3.22 kcal/g) from Nestlé® (Nantes, France), available *ad libitum*.

After the first 13 weeks, each dietary group received daily oral supplements for the additional 13 weeks. The Control groups (C) were administered mineral oil, the VD groups received 600 IU/kg/day of cholecalciferol (NeoBiotech, Xi'an, China), the ω3 groups were given 300 mg/kg/day of fish oil (18 % EPA/12 % DHA) from PhosphoTech Laboratoires® (Saint-Herblain, France), and the VD/ω3 groups received both cholecalciferol and fish oil. Animals were weighed at the beginning, the end, and once a week throughout the study.

The doses of vitamin D (600 IU/kg/day) and omega-3 fatty acids (300 mg/kg/day, 18 % EPA/12 % DHA) were chosen based on established human recommendations and prior preclinical studies. The vitamin D dose is within the range shown to achieve physiologically adequate plasma 25(OH)D levels in rats without risk of toxicity Chavhan et al., 2011, Kolieb et al., 2022, Shepard and Deluca, 1980. The omega-3 dose corresponds to commonly used ranges in rodent models (100–500 mg/kg/day) that have demonstrated beneficial metabolic and vascular effects (Adeyemi & Olayaki, 2017, 2018; Farooq et al., 2020; Iraz et al., 2005), ensuring effective supplementation in the context of diet-induced obesity.

### 2.3 Euthanasia

At the end of the supplementation period, the animals were anaesthetised by intraperitoneal injection of ketamine (60 mg/kg) and medetomidine (150 μg/kg). After the verification of good analgesia, animals were euthanized by exsanguination at the abdominal aorta for thoracic aorta extraction.

### 2.4 Preparation of aortic rings

Following euthanasia and exsanguination, the thoracic cavity of each rat was opened, and the thoracic aorta was excised after removing the heart-lung circulatory system. The aorta was immediately placed in a Krebs solution at 4 °C, aerated with carbogen (95 % O_2_; 5 % CO_2_) to maintain tissue viability. It was then cleaned of connective and perivascular tissues and cut into 3–4 mm rings.

### 2.5 Mounting and integrity of aortic rings

The aortic rings were mounted in organ bath chambers of emkaBATH4 devices (Emka Technologies®, Paris, France). Each chamber, with a 10 mL capacity, was equipped with two hooks: one fixed to support the ring and one movable attached to an isometric tension transducer, connected to an acquisition system to record contraction and relaxation movements. The chambers were filled with Krebs solution heated to 37 °C and bubbled with carbogen to maintain a physiological pH of 7.4.

An equilibration period of 1 h was conducted, during which the tension on the rings was gradually increased by 0.5 g every 15 min until reaching 2 g. This tension simulates the resting blood flow pressure.

Then, two viability tests were executed. Firstly, two successive KCl (8.10^−2^ M) tests were performed to verify vessel viability and restore membrane potentials. After each test, the chambers were rinsed until the baseline tension of 2 g was reestablished. Then, the rings were pre-contracted with a high concentration of Phe (10^−4^ M) to sensitize α-1 adrenergic receptors. The chambers were then rinsed until the baseline tension of 2 g was restored.

Finally, to check the endothelium integrity, we proceed to an endothelium test after a pre-contraction with Phe (3.10^−6^ M). Upon reaching a plateau, ACh (10^−6^ M) was added to induce endothelium-dependent relaxation. The endothelium was considered functional when the ACh-induced relaxation was ≥70 % compared to the Phe-induced contraction. The endothelial function test value was calculated using the following formula [1]:

[1] $$Endothelial\;test\;\left(\%\right)=100-\frac{Baseline\;Tension\;\left(g\right)-ACh\;Relaxation\;Tension\;\left(g\right)}{Baseline\;Tension\;\left(g\right)-Phe\;Contraction\;Tension\;\left(g\right)}\times 100$$

### 2.6 Experimental tests

Cumulative Concentration-Response Curves (CCRCs) to Ach (10^−9^ to 3.10^−5^ M) were performed after a pre-contraction period with Phe (3.10^−6^ M) for about 20 min. CCRCs were conducted without any additional molecules, in the presence of tempol (10^−3^ M), a Superoxide Dismutase (SOD) analogue antioxidant or in the presence of L-Arg (10^−3^ M), the eNOS substrate, and BH_4_ (10^−5^ M), an eNOS cofactor.

CCRCs to Phe (10^−9^ to 3.10^−5^ M) were also conducted in the presence or absence of AG (10^−4^ M), a selective inducible NOS (iNOS) inhibitor.

Finally, we also investigated the response to insulin (3.10^−6^ M) in pre-contracted rings, with or without Wort (10^−7^ M), a PI3K pathway inhibitor involved in insulin-mediated relaxation. The effect was observed for 30 min.

### 2.7 Statistical analysis

All data are expressed as mean ± Standard Error of the Mean (SEM). Relaxation is expressed as a percentage relative to the amplitude of the vasoconstriction induced by Phe, while contractions are reported as a percentage relative to the maximum response to KCl.

CCRCs were constructed using GraphPad Prism v9.0.0, and analysed with a nonlinear mixed-effects model using R software v4.3.1 (Thorin et al., 2010). The maximal effect (E_max_) and the half maximal effective concentration (EC_50_) were determined using the Hill equation [2]:

[2] $$E=\frac{E_{\max}\times C^{n}}{C^{n}+{\left[{EC}_{50}\right]}^{n}}$$

Where E is the effect observed, Emax is the maximal effect, C is the concentration, n is the Hill coefficient and EC_50_ is the concentration that produces 50 % of Emax.

The potency (pD_2_) value was calculated for CCRCs to Ach as follows [3]:

[3] $${pD}_{2}=-\log\left({EC}_{50}\right)$$

A p-value of less than 0.05 was considered statistically significant.

## 3 Results

### 3.1 Weight

**Fig. 1:**
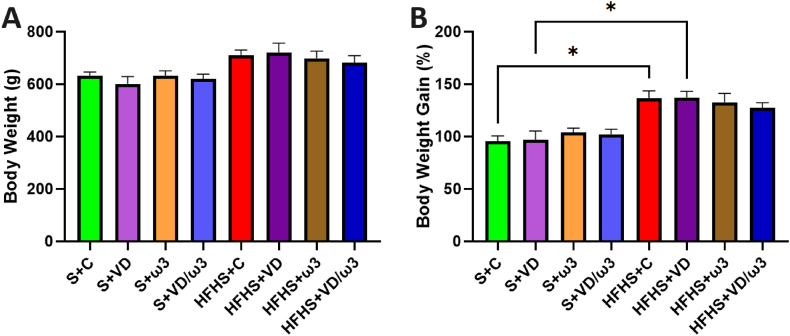
Body Weight at W26 and Body Weight Gain from Week 0 (W0) to Week 26 (W26). A: Body Weight (g) measured at W26. Values are expressed as mean ± SEM for each group (n = 6–8). B: Percentage body weight gain (%) calculated from W0 and W26. Values are expressed as mean ± SEM for each group. Statistical significance was determined using Kruskal-Wallis tests followed by post-hoc Dunn tests. ∗p < 0.05.

At the end of the study, there were no significant difference between groups in terms of body weight (Fig. 1A). However, the HFHS + C group exhibited a non-significant 12.23 % increase in body weight compared to the S + C group. Additionally, at W26, the HFHS+ω3 and HFHS + VD/ω3 groups showed increases of 10.47 % and 10.06 % respectively, compared to their corresponding standard groups.

Despite the lack of significant differences in final body weight, the evolution of weight throughout the study indicated a statistically significant increase in body weight gain in the HFHS + C and HFHS + VD groups compared to their respective standard group (p < 0.05) (Fig. 1B). These differences were not observed in the HFHS+ω3 and HFHS + VD/ω3 groups, indicating that these groups did not experience the same level of weight gain as the HFHS + C and HFHS + VD groups.

### 3.2 Cumulative Concentration-Response Curves to acetylcholine

**Fig. 2:**
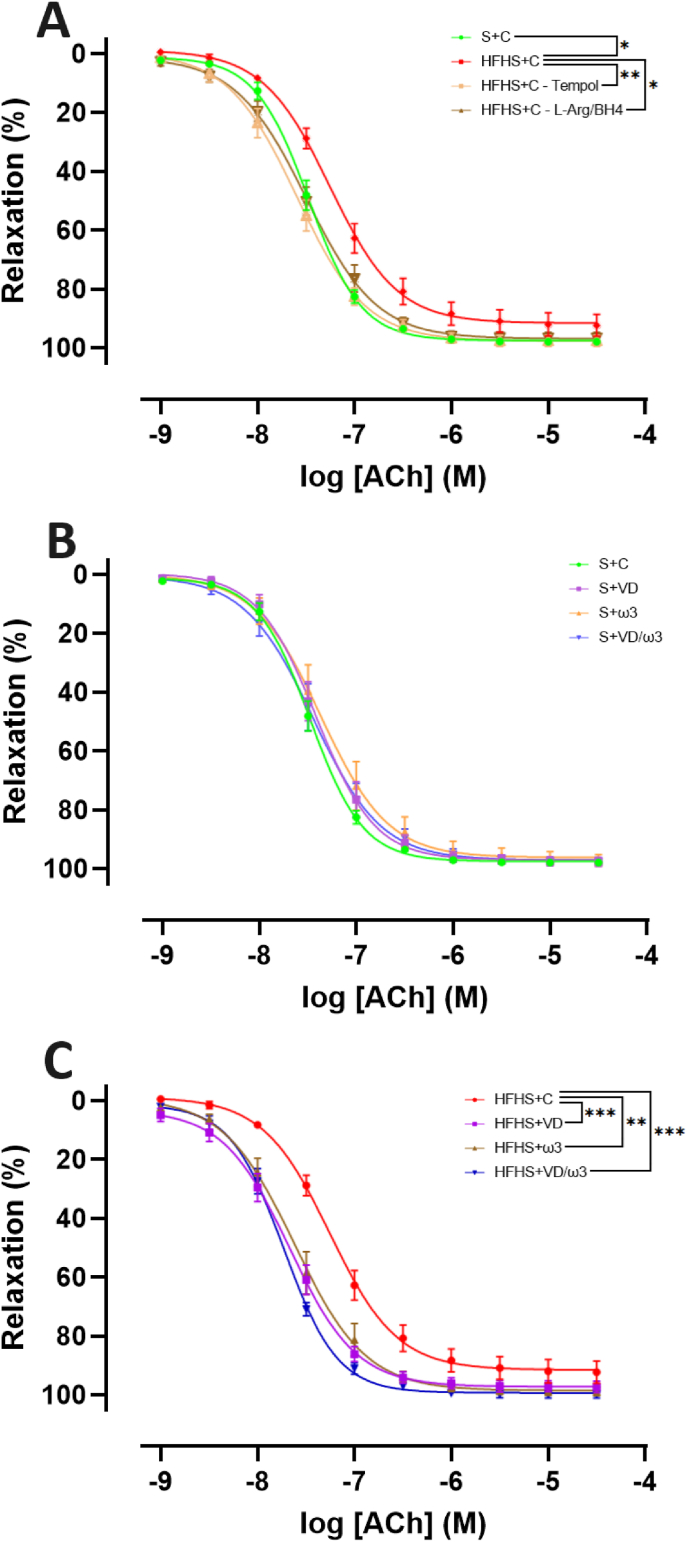
Cumulative Concentration-Response Curves (CCRCs) to acetylcholine (ACh; 10^−9^ to 3.10^−5^ M) were performed after a pre-contraction with phenylephrine (3.10^−6^ M). Values are expressed as mean ± SEM for each point, with n = 7–8 representing the number of aortic rings per group. A: CCRCs to Ach in the two Control (C) groups with Standard (S) or High-Fat High-Sugar diet (HFHS), and in the HFHS + C group following incubation with Tempol (10^−3^ M) or a combination of L-Arginine and tetrahydrobiopterin (L-Arg/BH4; 10^−4^ and 10^−5^ M, respectively). B: CCRCs to Ach in the four S groups supplemented with C, Vitamin D (VD), omega-3 fatty acids (ω3) or a combination of VD/ω3. C: CCRCs to Ach in the four HFHS groups supplemented with C, VD, ω3 or VD/ω3. CCRCs were constructed using a non-linear mixed effects model. Statistical significance for efficacy (E_max_) and potency (pD_2_) was determined using unpaired *t*-test or Kruskal-Wallis tests with post-hoc Dunn tests. Significant levels for pD2 are indicated as follows: ∗p < 0.05; ∗∗p < 0.01; ∗∗∗p < 0.001.

The construction of CCRCs to Ach showed a marked leftward shift in potency in the HFHS + C group compared to the S + C group (p < 0.01) (Fig. 2A). Although not statistically significant, the efficacy was also reduced in the HFHS + C group compared to the S + C group (E_max_ = 91.69 ± 3.57 % vs 97.34 ± 0.61 % respectively). Furthermore, a 2-h incubation with tempol significantly increased potency (p < 0.01), and a similar increase was observed with L-Arg/BH4 incubation (p < 0.05). These interventions restored normal Ach-mediated endothelium-dependent vasorelaxation, aligning their curve with the S + C curve.

In the S groups, CCRCs to Ach did not show any statistical differences. The potency and efficacy in the S + C (pD_2_ = 7.48 ± 0.02; E_max_ = 97.43 ± 0.95 %), S + VD (pD_2_ = 7.41 ± 0.04; E_max_ = 96.94 ± 1.56 %), S+ω3 (pD_2_ = 7.37 ± 0.06; E_max_ = 96.14 ± 2.36 %), S + VD/ω3 (pD_2_ = 7.45 ± 0.05; E_max_ = 97.00 ± 1.80 %) groups were identical (Fig. 2B).

In contrast, CCRCs to Ach in the HFHS groups revealed significant improvements in potency in the HFHS + VD (pD_2_ = 7.67 ± 0.04; p < 0.001), HFHS+ω3 (pD_2_ = 7.62 ± 0.05; p < 0.01) and HFHS + VD/ω3 (pD_2_ = 7.73 ± 0.02; p < 0.001) groups compared to the HFHS + C group (pD_2_ = 7.32 ± 0.06) (Fig. 2C). Although there was no statistical difference in E_max_, there was a trend towards higher efficacy in the HFHS + VD/ω3 group compared to the HFHS + S group (p = 0.076).

### 3.3 Cumulative Concentration-Response Curves to phenylephrine

**Fig. 3:**
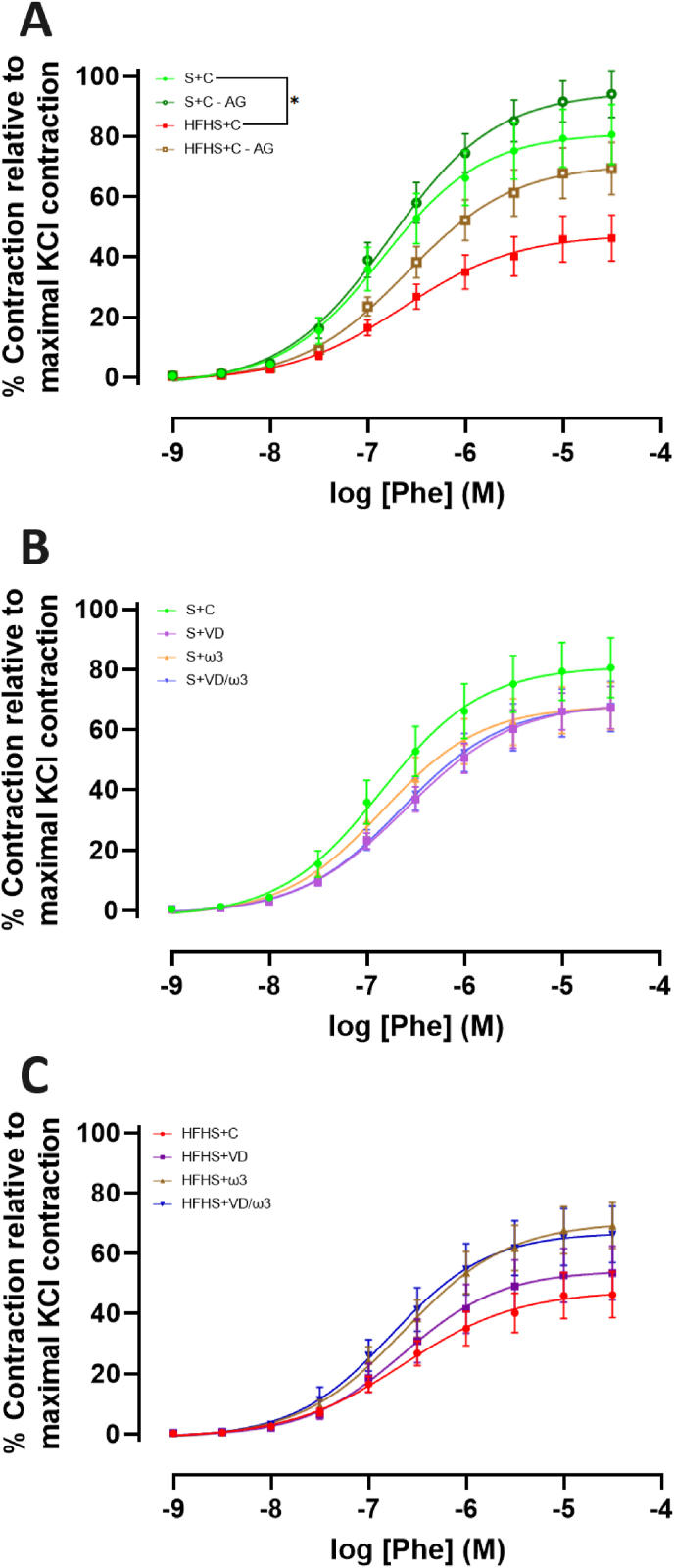
Cumulative Concentration-Response Curves (CCRCs) to Phenylephrine (Phe; 10^−9^ to 3.10^−5^ M). The response is expressed as the percentage increase in tension relative to the KCl-induced response. Values are expressed as mean ± SEM for each point, with n = 7–8 representing the number of aortic rings per group. A: CCRCs to Phe in the two Control (C) groups with Standard (S) or High-Fat High-Sugar diet (HFHS) and with or without incubation with Aminoguanidine (AG; 10^−4^ M). B: CCRCs to Phe in the four S groups supplemented with C, Vitamin D (VD), omega-3 fatty acids (ω3) or a combination of VD/ω3. C: CCRCs to Phe in the four HFHS groups supplemented with C, VD, ω3 or VD/ω3. CCRCs were constructed using a non-linear mixed effects model. Statistical significance for efficacy (E_max_) and potency (EC_50_) was determined using unpaired *t*-test or Kruskal-Wallis tests with post-hoc Dunn tests. Significant levels for Emax are indicated as follows: ∗p < 0.05.

The CCRCs to Phe revealed a significant decrease in the efficacy of contraction in the HFHS + C group compared to the S + C group (E_max_ = 47.52 ± 4.90 % vs. 81.11 ± 5.75 %, respectively; p < 0.05). Incubation with AG resulted in a non-significant increase in vasoconstriction in both groups, with AG restoring normal contraction in the HFHS + C group (Fig. 3A). There were no significant differences in potency between the groups.

In the S groups, CCRCs to Phe showed no statistical differences in potency or efficacy. The S + C, S + VD, S+ω3, and S + VD/ω3 groups displayed similar responses (Fig. 3B).

In the HFHS groups, no significant differences were observed in potency of efficacy. However, there was a trend towards increased vasoconstriction efficacy in the HFHS+ω3 and HFHS + VD/ω3 groups compared to the HFHS + C group (Fig. 3C).

### 3.4 Response to insulin

**Fig. 4:**
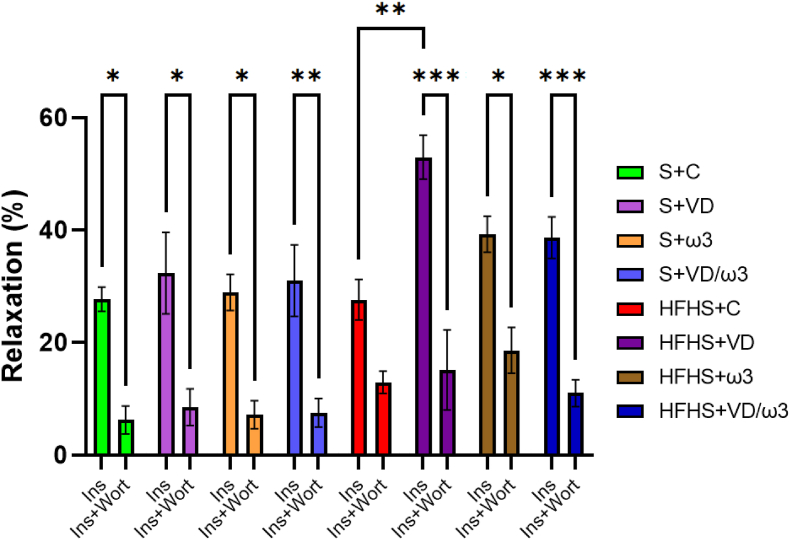
Maximal Relaxation to Insulin (Ins; 3.10^−6^ M) with or without incubation with Wortmannin (Wort; 10^−7^ M). Values are expressed as percentage of relaxation (%) after a pre-contraction with phenylephrine (3.10^−6^ M) and are represented as mean ± SEM, with n = 7–8 representing the number of aortic rings per group. ∗p < 0.05; ∗∗p < 0.01; ∗∗∗p < 0.001.

The injection of a high dose of insulin (3.10^−6^ M) induced significantly greater Ach-mediated endothelium-dependent vasorelaxation in the HFHS + VD group compared to the HFHS + C group (p < 0.01) (Fig. 4). Moreover, incubation with Wort led to a significant decrease in vasorelaxation in all groups except the HFHS + C group, which showed no significant difference in relaxation with or without incubation with Wort incubation (E_max_ = 12.91 ± 1.99 % vs. 27.62 ± 3.59 %, respectively).

## 4 Discussion

This study aimed to evaluate the impact of a 13-week supplementation with vitamin D and omega-3 on the tertiary prevention of vascular dysfunction associated with obesity in Wistar rats exposed to a 26-week HFHS diet. Endothelial function was evaluated by assessing both ACh-mediated and Ins-mediated vasorelaxation, processes that contribute to nitric oxide production via distinct signaling pathways.

Our study demonstrated that the HFHS + C group developed moderate obesity, with a 12 % weight gain compared to the S + C group, aligning with existing literature defining moderate obesity as a 10–25 % increase in body weight (Bagnol et al., 2012).

The analysis of ACh-mediated endothelium-dependent vasorelaxation demonstrated a significant decrease in potency in the HFHS + C group, suggesting endothelial dysfunction. Obesity is known to impair the NO pathway, which includes not only eNOS activity but also NO bioavailability and essential cofactors (Förstermann, 2010; Rashid, 2020). Studies have shown that a 24-week HFHS diet results in significantly higher levels of reactive oxygen species (ROS) (Apaijai et al., 2019). Additionally, a 12-week high-salt diet increased ROS production, leading to endothelial dysfunction in mice (Fu et al., 2018). Elevated glucose levels further exacerbate ROS production by activating NADPH oxidase, leading to O_2_^−•^ accumulation (Bonnefont-Rousselot, 2002; García-Prieto et al., 2015). This reacts with NO^•^ to form ONOO^−^, reducing NO^•^ bioavailability and impairing eNOS function (Förstermann and Münzel, 2006). Additionally, obesity-related inflammation further disrupts eNOS activity (Kwaifa et al., 2020). Collectively, these factors contribute to diminished endothelium-dependent vasorelaxation, highlighting oxidative stress and inflammation as key mechanisms of endothelial dysfunction in an HFHS diet.

The observed improvement in ACh-mediated vasorelaxation following tempol incubation strengthens the link between oxidative stress and HFHS diet-induced endothelial dysfunction, implying that ROS diminish NO^•^ bioavailability. Tempol, a SOD analogue, reduces O_2_^−•^ into hydrogen peroxide, thereby limiting its interaction with NO^•^ preventing the formation ONOO^−^, which depletes NO^•^ and impairs vasorelaxation (Beigrezaei and Nasri, 2017). Moreover, ONOO^−^ also oxidizes BH_4_, a crucial eNOS cofactor. Oxidation of BH_4_ into BH_2_ leads to eNOS uncoupling and further O_2_^−•^ production, worsening endothelial dysfunction (Münzel et al., 2008).

Similarly, the improvement in vasorelaxation after L-Arg/BH4 incubation suggests that a deficiency in these cofactors contributes to impaired NO^•^ synthesis. BH_4_ could enhances eNOS activity, while L-arginine might boost NO^•^ production. It has been shown that BH_4_ supplementation corrects eNOS dysfunction in insulin-resistant rats (Shinozaki et al., 2000). A 6-week supplementation of L-Arg improves vascular function in HFD-fed Wistar rats (Bogdański et al., 2015). By maintaining NO^•^ availability and eNOS function, these interventions counteract oxidative stress and endothelial impairment. Taken together, these results strongly support the central involvement of oxidative stress in HFHS-induced endothelial dysfunction.

The impaired Ach-mediated endothelium-dependent vasorelaxation underscores the detrimental impact of dietary-induced endothelial dysfunction, and our results confirms endothelial dysfunction in a 26-week HFHS diet rat model, despite not directly measuring ROS or enzyme levels. This highlights the necessity for strategies to mitigate oxidative stress and inflammation to preserve vascular health.

Despite the notable weight gain in obese rats, VD and ω3 supplementations did not significantly reduce weight although the HFHS + VD/ω3 group showed a slight trend toward better weight management. However, supplementation improved endothelium-dependent vasorelaxation, suggesting beneficial effects beyond weight regulation.

VD, ω3, and VD/ω3 supplementations significantly improved the potency of Ach-mediated endothelium-dependent vasorelaxation in HFHS-fed rats. This finding is consistent with previous findings showing that calcitriol incubation has been shown to enhance vascular function and reduce oxidative stress in aortic rings from diabetic Wistar rats (Sturza et al., 2019). VD and its analogues exert vasoprotective effects by reducing oxidative stress, inhibiting inflammation, and promoting vasodilation. Specifically, VD analogues lower NADPH oxidase levels in diabetic rats (Kono et al., 2013), while VD suppresses NF-κB signaling and pro-inflammatory cytokine production (Kim et al., 2020). Additionally, VD enhances eNOS transcription, boosting NO^•^ production to support vasodilation (Andrukhova et al., 2014).

Similarly, ω3 fatty acids have been reported to improve endothelial function by restoring eNOS expression in thoracic aorta of rats with chronic kidney disease (Zanetti et al., 2017). This improvement likely involves antioxidant and anti-inflammatory pathways, including reduced NADPH oxidase expression, ONOO^−^ production, and iNOS expression in aortic segments of HFD-diabetic rat models (Zhang et al., 2013). Furthermore, ω3 fatty activates PPAR proteins, suppressing NF-κB signaling (Grygiel-Górniak, 2014).

All these findings highlight the vascular protective role of VD and ω3 in counteracting HFHS-induced endothelial dysfunction through their antioxidant, anti-inflammatory, and eNOS-enhancing effects.

Our study demonstrated reduced phenylephrine-induced vasoconstriction in the HFHS + C group, indicating aortic hypocontractility likely linked to iNOS overexpression. In the context of obesity, elevated iNOS expression results in excessive NO^•^ production, which diminishes vascular smooth muscle sensitivity to vasoconstrictors and favors vasodilation (Shimizu et al., 2002).

To further investigate this phenomenon, we assessed the effects of AG, a selective iNOS inhibitor (Nilsson, 1999), known to mitigate NO^•^ overproduction associated with inflammation (Cinelli et al., 2020). Our results indicated that AG treatment increased the efficacy of Phe-induced contraction, although this improvement was not statistically significant. This aligns with previous studies where iNOS inhibition improved vascular reactivity (Gunnett et al., 2005). HFD models similarly exhibit impaired vasoconstriction due to iNOS overexpression (Silva et al., 2016), supporting the hypothesis that hypocontractility in our study results from iNOS overactivation. Given that excessive NO^•^ production by iNOS can disrupt vascular tone and contribute to endothelial dysfunction, these findings suggest that inflammation-driven iNOS upregulation may play a role in the altered vasoreactivity observed in HFHS-fed rats. However, as iNOS expression was not directly assessed in our study, further research is needed to confirm its contribution to endothelial dysfunction and vascular hypocontractility.

Nutritional supplementation had no impact on aortic rings from control rats. However, there were trends toward increased vasoconstriction efficacy in the HFHS+ω3 and HFHS + VD/ω3 groups compared to the HFHS + C group, although not statistically significant. This may be due to the ability of ω3 fatty acids to reduce oxidative stress and to inhibit iNOS activity (Zhang et al., 2013), potentially restoring vascular contraction. In contrast, the HFHS + VD group did not exhibit any impact on Phe-mediated vasoconstriction, suggesting that VD alone has a lesser influence on vascular contractility than ω3 or its combination with VD.

Obesity and insulin resistance significantly impair endothelial function by disrupting key signaling pathways. Normally, insulin induces vasorelaxation *via* the PI3K/Akt pathway, which activates eNOS and promotes NO^•^ production, leading to vasodilation (Pantazi et al., 2022). However, insulin also activates the MAPK/Endothelin-1 pathway, which promotes vasoconstriction (Pantazi et al., 2022). In the context of obesity, insulin resistance compromises the PI3K/Akt signaling, while enhancing endothelin-1 secretion, leading to a net effect favoring vasoconstriction and endothelial dysfunction (Muniyappa and Sowers, 2013).

Interestingly, our findings showed that only the HFHS + VD group displayed enhanced maximal insulin-induced relaxation relative to the HFHS + C group, suggesting that VD supplementation may enhance insulin sensitivity and restore endothelial function, potentially through activation of the PI3K/Akt pathway and increased NO^•^ production. VD is thought to enhance insulin secretion, receptor expression, and exocytosis (Maestro et al., 2000, 2002; Szymczak-Pajor et al., 2020), which may help counteract obesity-related insulin resistance and promote improved vasodilatory responses to insulin.

Wortmannin, a specific PI3K inhibitor, impairs insulin-mediated vasorelaxation by reducing NO^•^ production, as seen in studies on obese Zucker aortas (Yang et al., 2007). Our study revealed a marked reduction in insulin-mediated vasorelaxation across all groups, except the HFHS + C group, where Wortmannin had no significant impact. This indicates that in the untreated HFHS condition, the PI3K pathway is likely already compromised, rendering insulin-induced vasorelaxation largely independent of this signaling mechanism.

HFHS diet-induced obesity likely impairs PI3K signaling by reducing its expression or activity, thereby contributing to endothelial insulin resistance. This dysfunction is further amplified by obesity-associated oxidative stress and chronic inflammation, which together disrupt endothelial homeostasis and exacerbate PI3K pathway impairment, ultimately blunting insulin-mediated vasorelaxation.

Our study presents certain limitations, notably the lack of direct assessments of eNOS, iNOS, ONOO^−^, and O_2_^−•^ levels, as well as inflammatory markers, which restricts mechanistic interpretation. Furthermore, the relatively small sample size may constrain the broader applicability of our findings.

A 26-week HFHS diet induced endothelial dysfunction in the thoracic aorta of male Wistar rats, evidenced by diminished Phe-induced contraction and impaired insulin-mediated vasorelaxation. Although not directly measured, this dysfunction likely involved chronic inflammation, oxidative stress, reduced eNOS activity, impaired NO bioavailability, disrupted PI3K signaling, and elevated iNOS expression. Nutritional supplementation with VD, ω3, or both improved endothelial function: ω3 enhanced vasoconstriction, while VD restored insulin-mediated relaxation by improving insulin sensitivity. However, combining VD and ω3 did not produce an additive or synergistic effect beyond the improvements observed with each supplement individually. This lack of additional benefit may result from overlapping mechanisms, as both nutrients influence common pathways such as inflammation and oxidative stress, thereby limiting the potential for further enhancement. Alternatively, the doses or duration may have been insufficient to elicit synergism, or a ceiling effect may have occurred, whereby maximal endothelial improvement is already achieved with either nutrient individually. Such variable outcomes of VD and ω3 co-supplementation have been reported in previous studies (Basyreva et al., 2021; Valle et al., 2021).

In conclusion, a 13-week intervention with VD, ω3, or their combination improved HFHS-induced endothelial dysfunction. It might be hypothesized that these effects are mediated through mechanisms involving eNOS activity, reduction of oxidative stress, and modulation of inflammation.

Future studies should optimize VD and ω3 dosing to clarify their synergistic effects on endothelial dysfunction. Direct assessment of eNOS, iNOS, oxidative stress, and inflammation is needed to validate underlying mechanisms. Larger cohorts and clinical trials are essential to confirm the translational relevance of these interventions for obesity-related vascular complications.

## Author contributions

The authors’ responsibilities were as follows: Conceptualization: DLJ, JCD, EB, MYM; Data curation: DLJ, CT; Formal analysis: DLJ, CT; Investigation: DLJ, SD; Methodology: DLJ, SD; Resources: DLJ, SD; Supervision: JCD, EB, MYM; Validation: DLJ, MYM; Visualization: DLJ; Writing – original draft: DLJ, MYM; Writing – review and editing: All.

All authors have read and approved the final manuscript.

## Funding

This research did not receive any specific grant from funding agencies in the public, commercial, or not-for-profit sectors.

## Declaration of competing interest

The authors declare that they have no known competing financial interests or personal relationships that could have appeared to influence the work reported in this paper.

## Data Availability

Data will be made available on request.

## References

[bib1] Adeyemi W.J., Olayaki L.A. (2017). Effects of single or combined administration of salmon calcitonin and omega-3 fatty acids vs. diclofenac sodium in sodium monoiodoacetate- 437 induced knee osteoarthritis in male Wistar rats. J. Basic Clin. Physiol. Pharmacol..

[bib2] Adeyemi W.J., Olayaki L.A. (2018). Diclofenac – induced hepatotoxicity: low dose of omega-3 fatty acids have more protective effects. Toxicol. Rep..

[bib3] Andrukhova O., Slavic S., Zeitz U., Riesen S.C., Heppelmann M.S., Ambrisko T.D., Markovic M., Kuebler W.M., Erben R.G. (2014). Vitamin D is a regulator of endothelial nitric oxide synthase and arterial stiffness in mice. Mol. Endocrinol..

[bib4] Apaijai N., Arinno A., Palee S., Pratchayasakul W., Kerdphoo S., Jaiwongkam T., Chunchai T., Chattipakorn S.C., Chattipakorn N. (2019). High-saturated fat high-sugar diet accelerates left-ventricular dysfunction faster than high-saturated fat diet alone via increasing oxidative stress and apoptosis in obese-insulin resistant rats. Mol. Nutr. Food Res..

[bib5] Avogaro A., Kreutzenberg S.V. de, Fadini G. (2008). Endothelial dysfunction: causes and consequences in patients with diabetes mellitus. Diabetes Res. Clin. Pract..

[bib6] Bagnol D., Al-Shamma H.A., Behan D., Whelan K., Grottick A.J. (2012). Diet-Induced models of obesity (DIO) in rodents. Curr. Protoc. Neurosci..

[bib7] Basyreva L.Y., Vakhrusheva T.V., Letkeman Z.V., Maximov D.I., Fedorova E.A., Panasenko О.M. (2021). Effect of vitamin D3 in combination with Omega-3 polyunsaturated fatty acids on NETosis in type 2 diabetes mellitus patients. Oxid. Med. Cell. Longev..

[bib8] Beigrezaei S., Nasri H. (2017). Tempol as an antioxidant; an updated review on current knowledge. Ann. Res. Antioxid..

[bib9] Bogdański P., Suliburska J., Szulińska M., Sikora M., Walkowiak J., Jakubowski H. (2015). l-Arginine and vitamin C attenuate pro-atherogenic effects of high-fat diet on biomarkers of endothelial dysfunction in rats. Biomed. Pharmacother..

[bib10] Bonnefont-Rousselot D. (2002). Glucose and reactive oxygen species. Curr. Opin. Clin. Nutr. Metab. Care.

[bib11] Cai H., Harrison D.G. (2000). Endothelial dysfunction in cardiovascular diseases: the role of oxidant stress. Circ. Res..

[bib62] Chavhan S.G., Brar R.S., Banga H.S., Sandhu H.S., Sodhi S., Gadhave P.D., Kothule V.R., Kammon A.M. (2011). Clinicopathological studies on vitamin D3 toxicity and therapeutic evaluation of aloe vera in rats. Toxicol. Int..

[bib12] Chen J., Ye Z., Wang X., Chang J., Yang M., Zhong H., Hong F., Yang S. (2018). Nitric oxide bioavailability dysfunction involves in atherosclerosis. Biomed. Pharmacother..

[bib13] Cinelli M.A., Do H.T., Miley G.P., Silverman R.B. (2020). Inducible nitric oxide synthase: regulation, structure, and inhibition. Med. Res. Rev..

[bib14] Dyck M.C., Ma D.W., Meckling K.A. (2011). The anticancer effects of vitamin D and omega-3 PUFAs in combination via cod-liver oil: one plus one may equal more than two. Med. Hypotheses.

[bib15] Engin A., Engin A.B., Engin A. (2017). Obesity and Lipotoxicity.

[bib16] Farooq M.A., Gaertner S., Amoura L., Niazi Z.R., Park S.-H., Qureshi A.W., Oak M.-H., Toti F., Schini-Kerth V.B., Auger C. (2020). Intake of omega-3 formulation EPA:DHA 6:1 by old rats for 2 weeks improved endothelium-dependent relaxations and normalized the expression level of ACE/AT1R/NADPH oxidase and the formation of ROS in the mesenteric artery. Biochem. Pharmacol..

[bib17] Förstermann U. (2010). Nitric oxide and oxidative stress in vascular disease. Pflugers Arch. – Eur. J. Physiol..

[bib18] Förstermann U., Münzel T. (2006). Endothelial nitric oxide synthase in vascular disease. Circulation.

[bib19] Fu H., Chen J.-K., Lu W.-J., Jiang Y.-J., Wang Y.-Y., Li D.-J., Shen F.-M. (2018). Inflammasome-Independent NALP3 contributes to high-salt induced endothelial dysfunction. Front. Pharmacol..

[bib20] García-Prieto C.F., Hernández-Nuño F., Rio D.D., Ruiz-Hurtado G., Aránguez I., Ruiz-Gayo M., Somoza B., Fernández-Alfonso M.S. (2015). High-fat diet induces endothelial dysfunction through a down-regulation of the endothelial AMPK–PI3K–Akt–eNOS pathway. Mol. Nutr. Food Res..

[bib21] Grygiel-Górniak B. (2014). Peroxisome proliferator-activated receptors and their ligands: nutritional and clinical implications - a review. Nutr. J..

[bib22] Gunnett C.A., Lund D.D., McDowell A.K., Faraci F.M., Heistad D.D. (2005). Mechanisms of inducible nitric oxide synthase–mediated vascular dysfunction. Arterioscler. Thromb. Vasc. Biol..

[bib23] Hotamisligil G.S. (2006). Inflammation and metabolic disorders. Nature.

[bib24] Infante M., Sears B., Rizzo A.M., Mariani Cerati D., Caprio M., Ricordi C. (2020). Omega-3 PUFAs and vitamin D co-supplementation as a safe-effective therapeutic approach for core symptoms of autism spectrum disorder: case report and literature review. Nutr. Neurosci..

[bib25] Iraz M., Erdogan H., Ozyurt B., Ozugurlu F., Ozgocmen S., Fadillioglu E. (2005). Omega-3 essential Fatty acid supplementation and erythrocyte oxidant/Antioxidant status in rats. Ann. Clin. Lab. Sci..

[bib26] Kim D.-H., Meza C.A., Clarke H., Kim J.-S., Hickner R.C. (2020). Vitamin D and endothelial function. Nutrients.

[bib61] Kolieb E., Maher S.A., Shalaby M.N., Alsuhaibani A.M., Alharthi A., Hassan W.A., Et-Sayed K. (2022). Vitamin D and swimming exercise prevent obesity in rats under a high-fat diet via targeting FATP4 and TLR4 in the liver and adipose tissue. Int. J. Environ. Res. Public Health.

[bib27] Kono K., Fujii H., Nakai K., Goto S., Kitazawa R., Kitazawa S., Shinohara M., Hirata M., Fukagawa M., Nishi S. (2013). Anti-Oxidative effect of vitamin D analog on incipient vascular lesion in non-obese type 2 diabetic rats. Am. J. Nephrol..

[bib28] Konukoglu D., Uzun H., Islam MdS. (2017). Hypertension: from Basic Research to Clinical Practice.

[bib29] Kwaifa I.K., Bahari H., Yong Y.K., Noor S.M. (2020). Endothelial dysfunction in obesity-induced inflammation: molecular mechanisms and clinical implications. Biomolecules.

[bib30] Maestro B., Campión J., Dávila N., Calle C. (2000). Stimulation by 1, 25-Dihydroxyvitamin D3 of insulin receptor expression and insulin responsiveness for glucose transport in U-937 human promonocytic cells. Endocr. J..

[bib31] Maestro B., Molero S., Bajo S., Dávila N., Calle C. (2002). Transcriptional activation of the human insulin receptor gene by 1,25-dihydroxyvitamin D3. Cell Biochem. Funct..

[bib32] Moura-Assis A., Afonso M.S., de Oliveira V., Morari J., dos Santos G.A., Koike M., Lottenberg A.M., Ramos Catharino R., Velloso L.A., Sanchez Ramos da Silva A., de Moura L., Ropelle E.R., Pauli J.R., Cintra D.E.C. (2018). Flaxseed oil rich in omega-3 protects aorta against inflammation and endoplasmic reticulum stress partially mediated by GPR120 receptor in Obese, diabetic and dyslipidemic mice models. J. Nutr. Biochem..

[bib33] Mozos I., Marginean O. (2015). Links between vitamin D deficiency and cardiovascular diseases. BioMed Res. Int..

[bib34] Muniyappa R., Sowers J.R. (2013). Role of insulin resistance in endothelial dysfunction. Rev. Endocr. Metab. Disord..

[bib35] Münzel T., Sinning C., Post F., Warnholtz A., Schulz E. (2008). Pathophysiology, diagnosis and prognostic implications of endothelial dysfunction. Ann. Med..

[bib36] Nieto-Vazquez I., Fernández-Veledo S., Krämer D.K., Vila-Bedmar R., Garcia-Guerra L., Lorenzo M. (2008). Insulin resistance associated to obesity: the link TNF-alpha. Arch. Physiol. Biochem..

[bib37] Nilsson B.-O. (1999). Biological effects of aminoguanidine: an update. Inflamm. Res..

[bib38] Pantazi K., Karlafti E., Bekiaridou A., Didagelos M., Ziakas A., Didangelos T. (2022). Insulin receptors and insulin action in the heart: the effects of left ventricular assist devices. Biomolecules.

[bib39] Rashid H. (2020). Prebiotics supplementation ameliorates high fat high sugar diet-associated oxidative stress. Pak. Vet. J..

[bib40] Refaat B., Abdelghany A.H., Ahmad J., Abdalla O.M., Elshopakey G.E., Idris S. (2022). Vitamin D3 enhances the effects of omega-3 oils against metabolic dysfunction-associated fatty liver disease in rat. Biofactors.

[bib41] Renke G., Starling-Soares B., Baesso T., Petronio R., Aguiar D., Paes R. (2023). Effects of vitamin D on cardiovascular risk and oxidative stress. Nutrients.

[bib42] Said M.A. (2022). Vitamin D attenuates endothelial dysfunction in streptozotocin induced diabetic rats by reducing oxidative stress. Arch. Physiol. Biochem..

[bib60] Shepard R.M., Deluca H.F. (1980). Plasma concentrations of vitamin D3 and its metabolites in the rat as influenced by vitamin D3 or 25-hydroxyvitamin D3 intakes. Arch. Biochem. Biophys..

[bib43] Shimizu T., Sakamoto A., Ogawa R. (2002). Inhibition of inducible nitric oxide synthase attenuates Interleukin-1β induced vascular hyporeactivity in the rabbit. J. Nippon Med. Sch..

[bib44] Shinozaki K., Nishio Y., Okamura T., Yoshida Y., Maegawa H., Kojima H., Masada M., Toda N., Kikkawa R., Kashiwagi A. (2000). Oral administration of tetrahydrobiopterin prevents endothelial dysfunction and vascular oxidative stress in the aortas of insulin-resistant rats. Circ. Res..

[bib45] Silva J.F., Correa I.C., Diniz T.F., Lima P.M., Santos R.L., Cortes S.F., Coimbra C.C., Lemos V.S. (2016). Obesity, inflammation, and exercise training: relative contribution of iNOS and eNOS in the modulation of vascular function in the mouse aorta. Front. Physiol..

[bib46] Sitia S., Tomasoni L., Atzeni F., Ambrosio G., Cordiano C., Catapano A., Tramontana S., Perticone F., Naccarato P., Camici P., Picano E., Cortigiani L., Bevilacqua M., Milazzo L., Cusi D., Barlassina C., Sarzi-Puttini P., Turiel M. (2010). From endothelial dysfunction to atherosclerosis. Autoimmun. Rev..

[bib47] Sturza A., Văduva A., Uțu D., Rațiu C., Pop N., Duicu O., Popoiu C., Boia E., Matusz P., Muntean D.M., Olariu S. (2019). Vitamin D improves vascular function and decreases monoamine oxidase A expression in experimental diabetes. Mol. Cell. Biochem..

[bib48] Szymczak-Pajor I., Drzewoski J., Śliwińska A. (2020). The molecular mechanisms by which vitamin D prevents insulin resistance and associated disorders. Int. J. Mol. Sci..

[bib49] Thorin C., Mallem M.Y., Noireaud J., Gogny M., Desfontis J.-C. (2010). Nonlinear mixed effects models applied to cumulative concentration–response curves. J. Pharm. Pharmacol..

[bib50] Valle M., Mitchell P.L., Pilon G., St-Pierre P., Varin T., Richard D. (2021). Cholecalciferol supplementation does not prevent the development of metabolic syndrome or enhance the beneficial effects of Omega-3 fatty acids in Obese mice. J. Nutr..

[bib51] Veloudi P., Jones G., Sharman J.E. (2016). Effectiveness of vitamin D supplementation for cardiovascular health outcomes. Pulse.

[bib52] Wall R., Ross R.P., Fitzgerald G.F., Stanton C. (2010). Fatty acids from fish: the anti-inflammatory potential of long-chain omega-3 fatty acids. Nutr. Rev..

[bib53] Wamberg L., Pedersen S.B., Rejnmark L., Richelsen B. (2015). Causes of vitamin D deficiency and effect of vitamin D supplementation on metabolic complications in obesity: a review. Curr. Obes. Rep..

[bib54] Wang L., Cheng C.K., Yi M., Lui K.O., Huang Y. (2022). Targeting endothelial dysfunction and inflammation. J. Mol. Cell. Cardiol..

[bib55] Wang Q., Liang X., Wang L., Lu X., Huang J., Cao J., Li H., Gu D. (2012). Effect of omega-3 fatty acids supplementation on endothelial function: a meta-analysis of randomized controlled trials. Atherosclerosis.

[bib56] Yang A.-L., Chao J.-I., Lee S.-D. (2007). Altered insulin-mediated and insulin-like growth factor-1-mediated vasorelaxation in aortas of obese Zucker rats. Int. J. Obes..

[bib57] Zanetti M., Gortan Cappellari G., Barbetta D., Semolic A., Barazzoni R. (2017). Omega 3 polyunsaturated Fatty acids improve endothelial dysfunction in chronic renal failure: role of eNOS activation and of oxidative stress. Nutrients.

[bib58] Zhang W., Fu F., Tie R., Liang X., Tian F., Xing W., Li J., Ji L., Xing J., Sun X., Zhang H. (2013). Alpha-linolenic acid intake prevents endothelial dysfunction in high-fat diet-fed streptozotocin rats and underlying mechanisms. Vasa.

[bib59] Zhu Y., Wen L., Wang S., Zhang K., Cui Y., Zhang C., Feng L., Yu F., Chen Y., Wang R., Ma X. (2021). Omega-3 fatty acids improve flow-induced vasodilation by enhancing TRPV4 in arteries from diet-induced obese mice. Cardiovasc. Res..

